# Differences in the bacteriome of swab, saliva, and tissue biopsies in oral cancer

**DOI:** 10.1038/s41598-020-80859-0

**Published:** 2021-01-13

**Authors:** Divya Gopinath, Rohit Kunnath Menon, Chong Chun Wie, Moinak Banerjee, Swagatika Panda, Deviprasad Mandal, Paresh Kumar Behera, Susanta Roychoudhury, Supriya Kheur, Michael George Botelho, Newell W. Johnson

**Affiliations:** 1 Faculty of Dentistry, Prince Philip Dental Hospital, University of Hong Kong, Street 34 Hospital Rd, Sai Ying Pun, Hong Kong, SAR China; 2 Oral Diagnostic and Surgical Sciences, School of Dentistry, International Medical University, Kuala Lumpur, Malaysia; 3 Clinical Dentistry, School of Dentistry, International Medical University, Kuala Lumpur, Malaysia; 4 School of Pharmacy, Monash University, Selangor, Malaysia; 5 Human Molecular Genetics Lab, Rajiv Gandhi Centre for Biotechnology, Trivandrum, India; 6 Department of Oral Pathology and Microbiology, Siksha O Anusandhan University, Bhubaneswar, India; 7 Head and Neck Oncology, Acharya Harihara Regional Cancer Centre, Bhubaneswar, India; 8 Saroj Gupta Cancer Centre and Research Institute, Kolkata, India; 9 Department of Oral Pathology and Microbiology, D.Y. Patil Dental College, D.Y. Patil Vidyapeeth, Pune, Maharashtra India; 10 Menzies Health Institute Queensland, School of Dentistry and Oral Health, Griffith University, Brisbane, QLD Australia; 11 Faculty of Dentistry, Oral and Craniofacial Sciences, King’s College London, London, UK

**Keywords:** Cancer, Head and neck cancer, Oral cancer, Microbial communities, Metagenomics, Microbial ecology, Microbiome

## Abstract

Microbial dysbiosis has been implicated in the pathogenesis of oral cancer. We analyzed the compositional and metabolic profile of the bacteriome in three specific niches in oral cancer patients along with controls using 16SrRNA sequencing (Illumina Miseq) and DADA2 software. We found major differences between patients and control subjects. Bacterial communities associated with the tumor surface and deep paired tumor tissue differed significantly. Tumor surfaces carried elevated abundances of taxa belonging to genera *Porphyromonas, Enterobacteriae, Neisseria, Streptococcus* and *Fusobacteria,* whereas *Prevotella, Treponema, Sphingomonas, Meiothermus* and *Mycoplasma* genera were significantly more abundant in deep tissue. The most abundant microbial metabolic pathways were those related to fatty-acid biosynthesis, carbon metabolism and amino-acid metabolism on the tumor surface: carbohydrate metabolism and organic polymer degradation were elevated in tumor tissues. The bacteriome of saliva from patients with oral cancer differed significantly from paired tumor tissue in terms of community structure, however remained similar at taxonomic and metabolic levels except for elevated abundances of *Streptococcus, Lactobacillus* and *Bacteroides,* and acetoin-biosynthesis, respectively. These shifts to a pro-inflammatory profile are consistent with other studies suggesting oncogenic properties. Importantly, selection of the principal source of microbial DNA is key to ensure reliable, reproducible and comparable results in microbiome studies.

## Introduction

Despite significant advances in techniques for early diagnosis and management, oral cancer remains a major cause of morbidity and mortality in many parts of the world. Oral cavity cancers, principally comprising squamous cell carcinomas of the lip, tongue and mouth, collectively were estimated to account for 355,000 diagnoses and over 177,000 deaths in 2018^1^. There is a wide disparity in the incidence rates of oral cancer across the world, which is largely explained by differences in lifestyle^1–3^. The burden of oral cancer is highest in the Indian subcontinent, accounting for one-third of the total global burden^2^.

Well-recognized lifestyle risk factors for oral cancer include use of tobacco (both smoked and smokeless), areca nut, excessive alcohol intake, diets lacking in antioxidant vitamins and minerals; more recently poor oral hygiene and periodontitis are emerging as significant cofactors^4,5^. These factors modify the microflora in particular niches in the oral cavity ^6–9^ and as such they and their metabolites act as environmental risk modifiers for the initiation and progression of oral cancer. The interaction between microbial communities and their host, in many biological niches, has been found to be functionally involved in health, and the pathogenesis of numerous diseases^10,11^. Links between specific bacteria and oral carcinogenesis have been reported in animal studies^12–14^. However, such associations in humans are less clear and are confounded by the inability to separate organisms having a primary role in aetiopathogenesis (“drivers”) from those who have colonized at a later stage (“passengers”)^15,16^. The relationship between the microbiome and oral cancer appears multifaceted; specific microbes may play some role in initiation and progression of a tumor or functional dysbiosis of the entire microbial ecosystem may contribute to the complex events of carcinogenesis and disease progression^11,14^.

There has been a recent surge in studies examining the association between the oral microbiome and ‘Head and Neck Cancer’ using 16S rRNA gene sequencing^12–16^. However, consistent conclusions have not been reached because of variation in the methods for bacterial profiling. Also, there are inconsistencies in samples used (fresh or archival frozen samples), type of controls (adjacent mucosa/contralateral mucosa/healthy people/people with benign mucosal lesions), types of samples collected (swab or tissue or saliva), histological type of cancer, and anatomical subsites involved: indeed oral, pharyngeal and laryngeal sites are, regrettably, often combined^11^. Head and neck subsites differ at clinical and molecular levels and have different prognoses^17–20^. Microbial impacts on host gene expression in mammals have been shown to be extremely site-specific^21,22^. Research has revealed considerable variations in microbiome composition at most body sites which have varied across countries and ethnicities: hence there is a need to investigate geographic and ethnic variations in the oral microbiome^23,24^. Published studies of the microbiomics of head and neck cancer have so far come from few countries^16,25^. Geographical variations in the microbial community have been attributed to dissimilarities in behavioral factors including diet, substance use, hygiene and host factors including genetics and immunity^26^. Therefore, understanding the bacteriome associated with oral cancer in South Asian cohorts where the disease persists in epidemic proportions is of utmost significance.

The role of the bacteriome in a particular environment is linked to its overall metabolic activity^27,28^. Changes to the tumor microenvironment elicited by bacterial co-metabolism can affect the host immune response and have effects on cancer therapeutics^28,29^. Most studies so far have made functional inferences based on operational taxonomic unit (OTU) assignments to published genomes^30^. However, there are many bacterial genomes in databases derived from 16S rRNA amplification which harbor genes with analogous or overlapping functions. In this current study, for the first time in relation to the oral microbiota, we have used the phylogenetic tree placement method to study the bacteriome of oral squamous cell carcinoma (OSCC) patients by utilizing a consensus genome constructed with all genomes shared by members of the same clade originating from each node, thus increasing the resolution of the inferred metabolic pathways^30^. This is very constructive as it can lead to identifying the most relevant metabolic pathways of a bacterial community in the tumor microenvironment.

## Results

### Study population and sample characteristics

The case and control cohorts were predominantly male with a mean age of 49.31 ± 13.24 years for cancers and 50.67 ± 6.81 years for controls. The demographic characteristics of subjects are provided in Supplementary Material (Supplementary Table 1). A total of 48 cases and 46 controls were sampled. A subset of 44 matched surface swab and deeper tissues from OSCC patients were utilized for comparison between mucosal bacteriome and tissue-associated microbiome. A subset of 25 matched tissue and whole mouth fluid (WMF) samples from OSCC patients were utilized for comparison between WMF and tissue-associated microbiomes.

### The structure and function of the bacteriome of oral cancer tissues differs significantly from that of healthy control tissue

**Figure 1:**
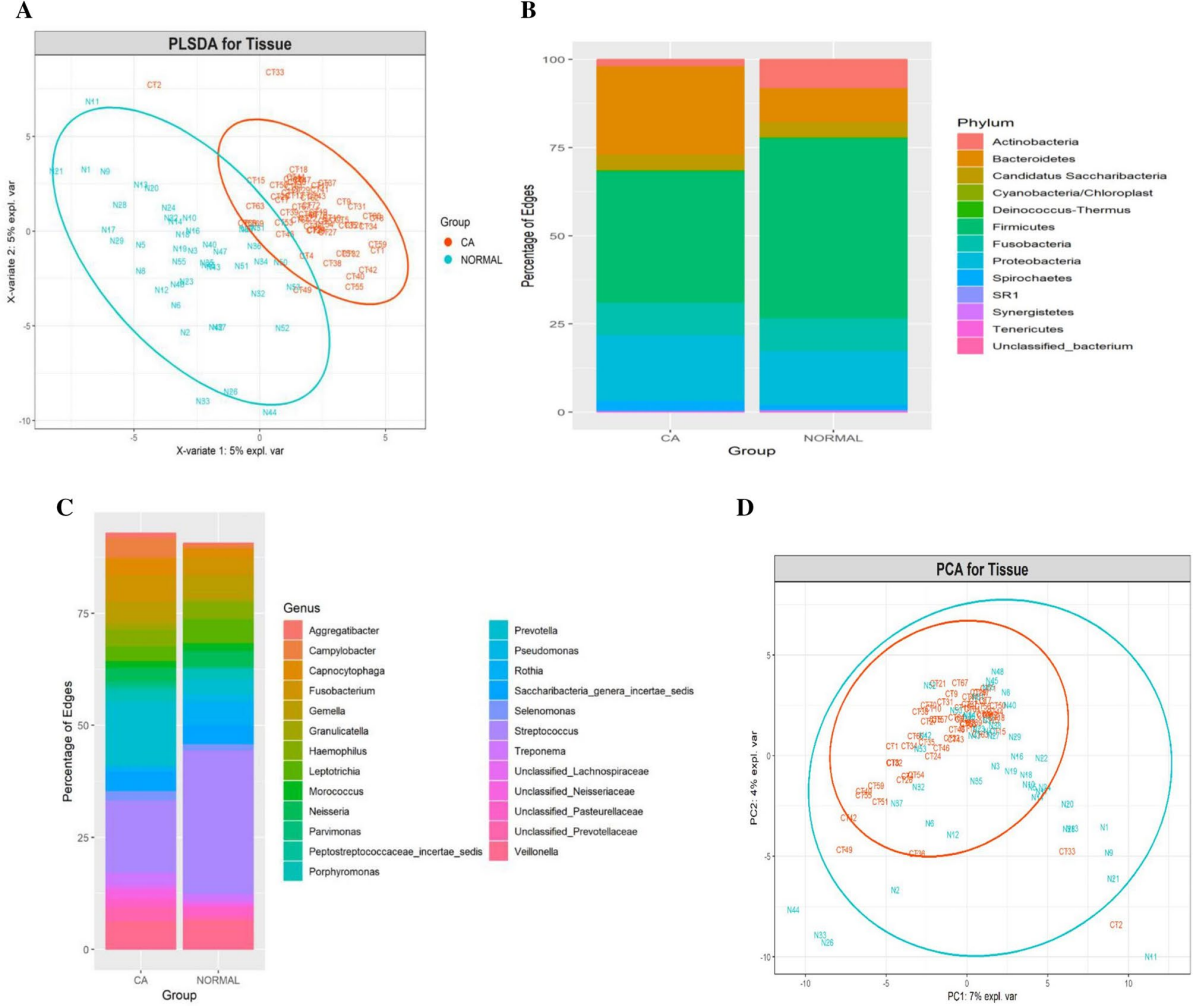
(**A)** PLS-DA plot of oral microbiota between the cancer (CA) tissues and normal tissue sample (P < 0.01). P value was determined by PERMANOVA. Percentage distribution of bacterial communities across samples in CA and normal tissues at the phylum **(B)** and genus **(C)** levels. (**D)** PCA plot.

Partial least square discriminant analysis (PLS-DA) and comparison of the abundance of bacterial taxa at phylum and genus revealed significant differences between the bacteriome associated with oral cancer tissue and normal healthy oral mucosa from controls. In PLS-DA, the cases and controls formed separate clusters; the results being statistically significant as assessed by PERMANOVA (P < 0.01). However, the cancer tissue bacteriomes were more closely clustered than those from the control tissues (Fig. 1A).

The bacteriome in the cancer tissue and the control tissue were examined at different taxonomic levels. We identified differences in the distribution of eight phyla and eighteen genera (Fig. 1B,C). The top three phyla in the cancer samples were: *Firmicutes, Bacteroides* and *Spirochetes*. In normal healthy control tissues, the predominant phylum was *Firmicutes* followed by *Spirochetes* and *Actinobacteria*. The cancer tissue demonstrated a decrease in the relative abundance of *Firmicutes* coupled with an increase in *Bacteroidetes* relative to controls. Also, *Actinobacteria* showed a decrease in abundance in the cancer tissues in comparison with the normal controls. We did not find any statistically significant associations between clinical variables including anatomic subsites, tumor size and nodal status and microbiomic profiles in cancer tissue. The PCA (principal component analysis) plot for the groups is illustrated in Fig. 1D.

**Figure 2:**
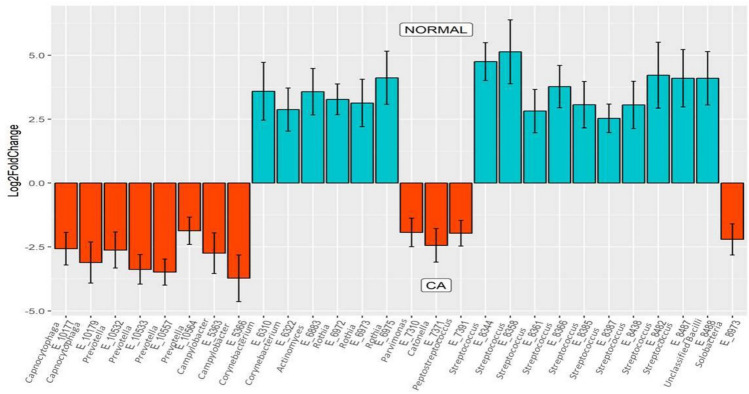
Differentially abundant taxa identified in cancer and normal tissues by DESeq2 analysis. Twenty-eight taxa were differentially abundant between the tumor surface and the tumor tissue.

**Figure 3:**
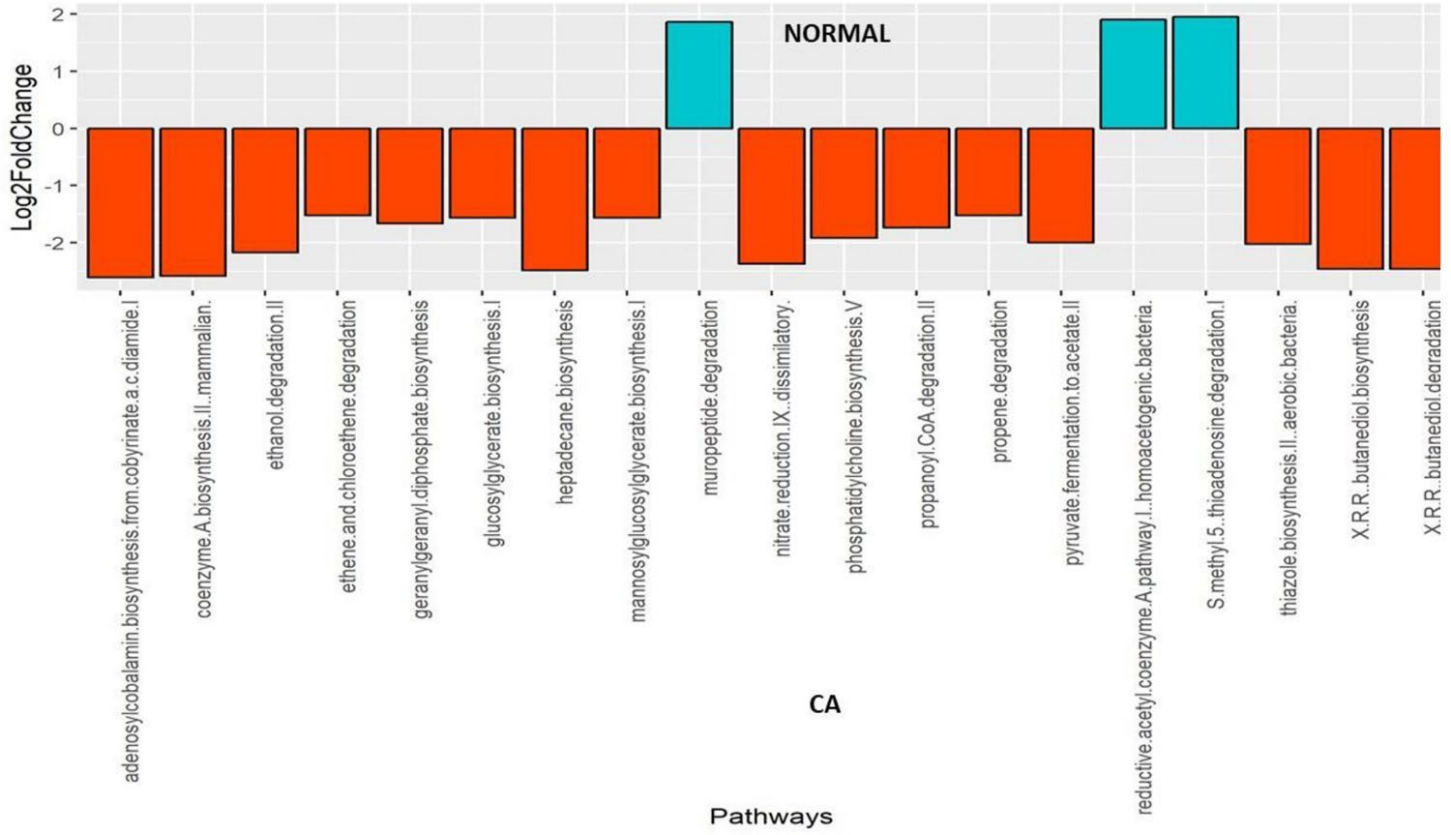
Differentially abundant metabolic pathways identified in cancer and normal tissues by DESeq2 Analysis. Increased abundance of sixteen bacterial metabolic pathways were present in tumor tissue relative to controls.

DESeq2 analysis revealed several taxa belonging to genera *Solobacteria, Peptostreptococcus, Catonella, Finegoldia, Campylobacter, Prevotella,* and *Capnocytophaga* with significantly higher abundances in the cancer tissues when compared to the tissues of control subjects (P < 0.01).The genera *Corynebacterium, Actinomyces, Rothia* and *Streptococcus* were significantly greater in the controls (P < 0.01) (Fig. 2). Nineteen metabolic pathways were found to be differentially abundant between cancer tissue bacteriome and normal tissue bacteriome (Fig. 3). The MA-plots for the DESeq2 analysis is provided in the supplementary material.

### Oral cancer bacteriome differs between matched tumor surface and deep tumor tissue samples

**Figure 4:**
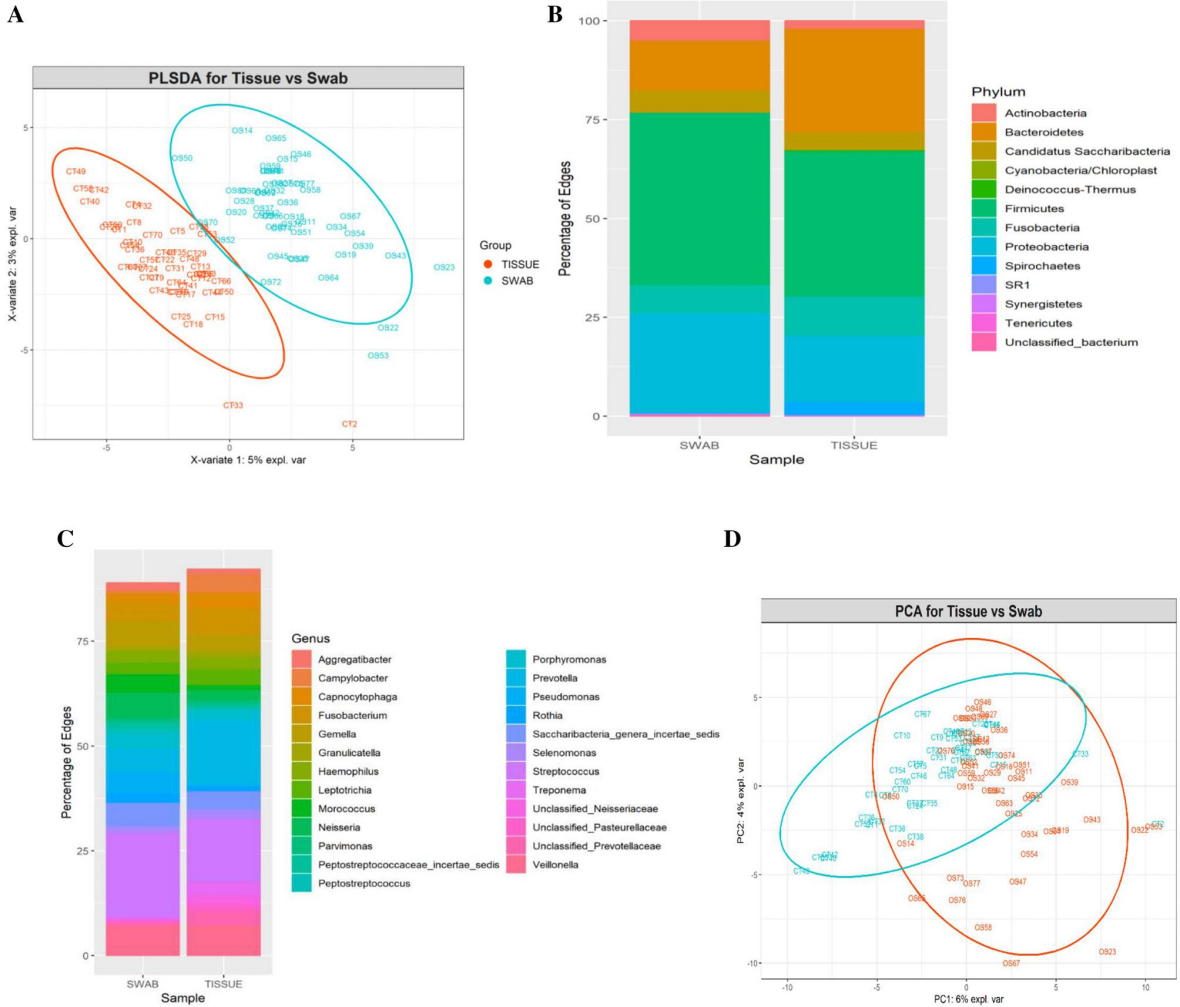
**(A)** PLS-DA plot of oral microbiota between matched cancer tissue and tumor surface samples in cancer patients, (P < 0.01) P value was determined by PERMANOVA. Percentage distribution of bacterial communities across samples at the phylum **(B)** and genus **(C)** level (**D)** PCA plot.

**Figure 5:**
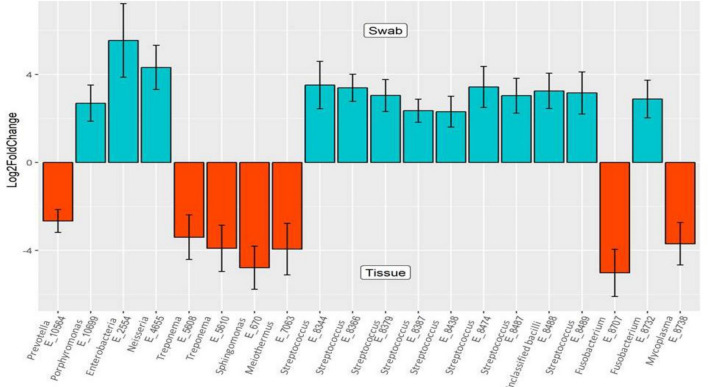
Differentially abundant taxa identified in cancer tissue and tumor surface groups by DESeq2 analysis. Twenty taxa were differentially abundant between the tumor surface and the tumor tissue.

**Figure 6:**
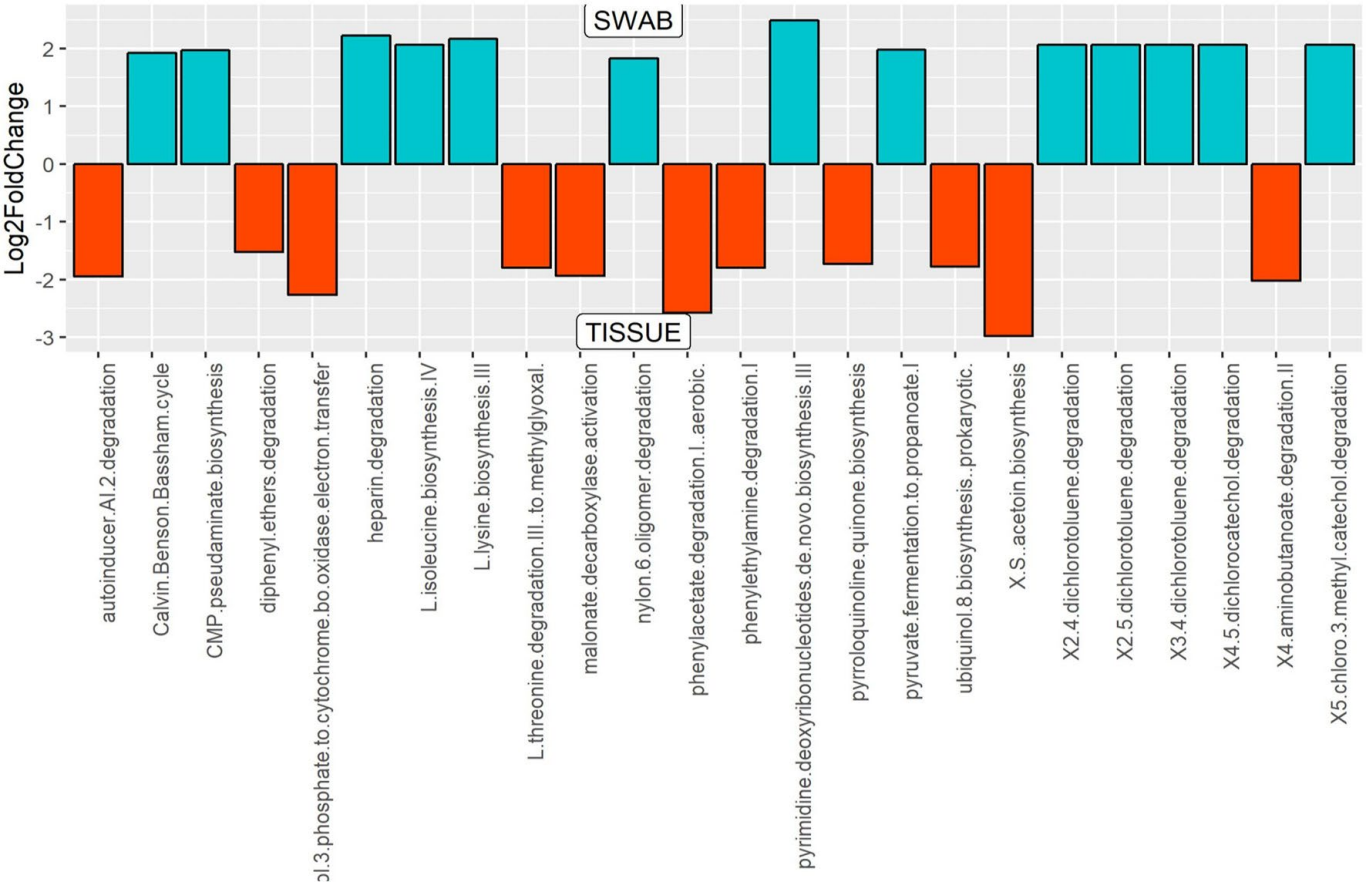
Differentially abundant metabolic pathways identified in cancer tissue and tumor surface by DESeq2 Analysis. Levels of twenty-four metabolic pathways were differentially abundant between the tumor surface and the tumor tissue.

The bacterial community of matched tumor surface mucosa and tumor tissue differed significantly, both at the community level (Fig. 4A) as well as at taxonomic levels (Fig. 4B,C). The PCA plot for the groups is illustrated in Fig. 4D. There was a significant difference in the abundance of phyla *Bacteroidetes* and *Fusobacteria* on tumor surfaces illustrating a lesser proportion of the former and higher proportion of the latter in comparison to matched cancer tissues. Especially, the abundances of taxa belonging to genera *Porphyromonas, Enterobacteriae, Neisseria, Streptococcus* and *Fusobacterium* were significantly elevated on the tumor surfaces: *Prevotella, Treponema, Sphingomonas, Meiothermus* and *Mycoplasma* genera were significantly more abundant in matched deep tumor tissue samples (Fig. 5). Twenty-four metabolic pathways were found to differ between tumor surface and deeper tissue (Fig. 6). The most abundant pathways were those related to fatty-acid biosynthesis, carbon metabolism and amino-acid metabolism on the tumor surface: carbohydrate metabolism and organic polymer degradation were elevated in cancer tissues.

### Differences in WMF and tumor tissue bacteriomes

**Figure 7:**
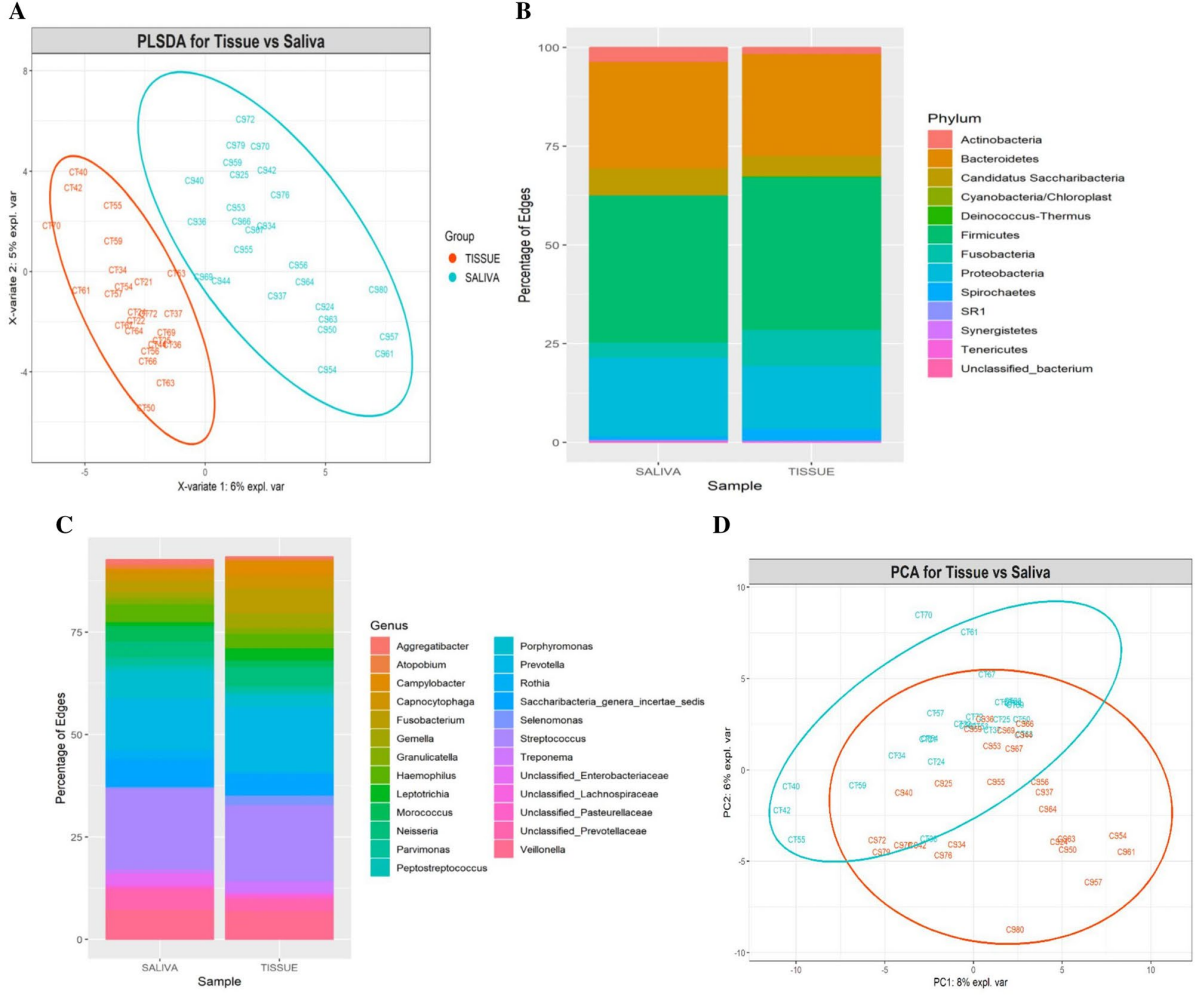
**(A)** Partial least square discriminant score plot of oral microbiota between the cancer tissue and WMF sample (P < 0.01). P value was determined by PERMANOVA. Percentage distributions of bacterial communities across samples at the phylum **(B)** and genus **(C)** levels (**D)** PCA plot.

**Figure 8:**
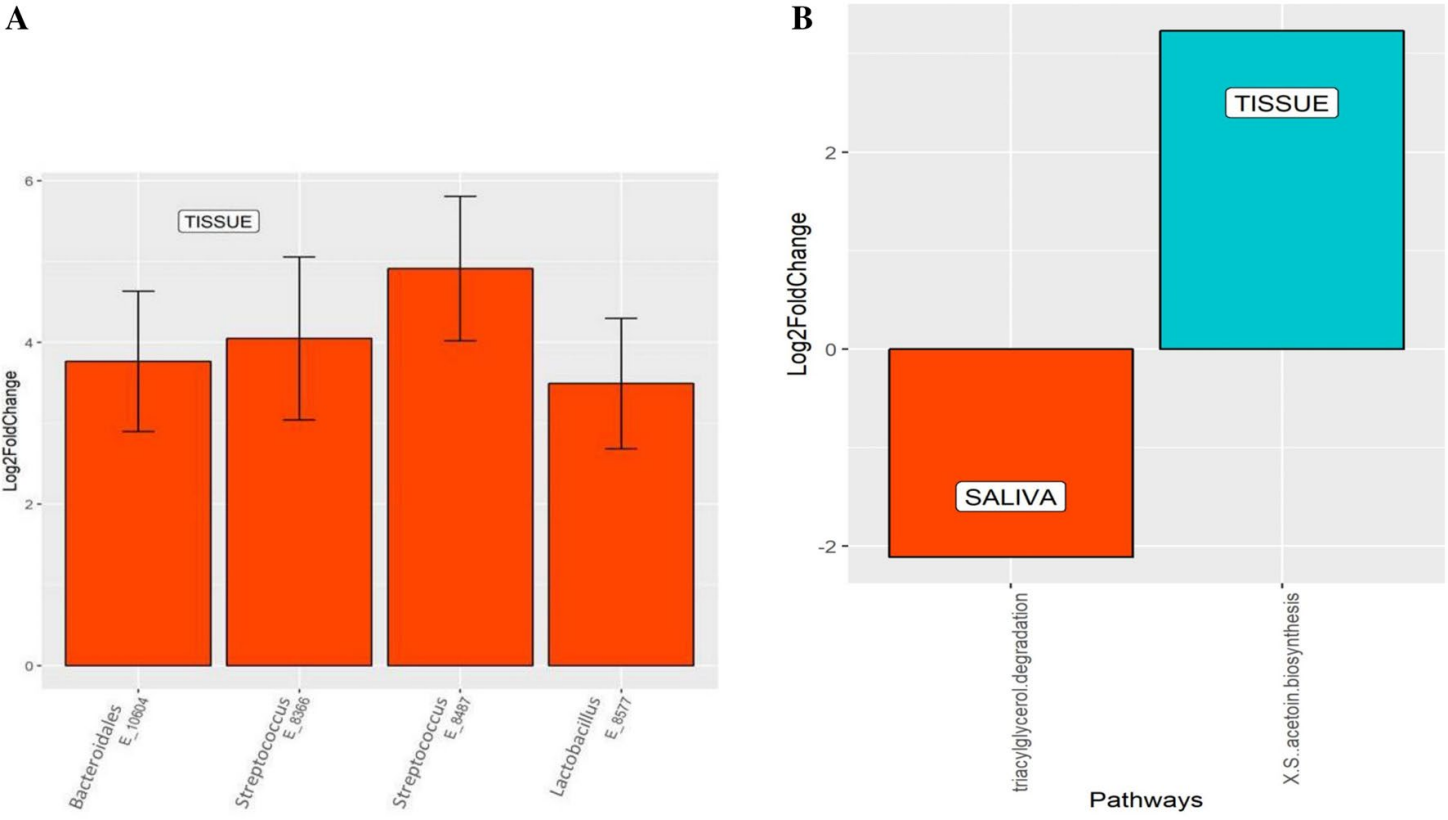
**(A)** Differential taxa identified in cancer tissue in comparison with WMF by DESeq2 Analysis. Four taxa were abundant in tumor tissue relative to WMF. (**B)** Differential pathways identified in cancer tissue and WMF groups by DESeq 2 Analysis. Two metabolic pathways namely trigylcerol degradation and acetoin biosynthesis were differentially abundant between the tumor surface and the tumor tissue.

Because of the potential of WMF, through its microbiota, to be a source of a biomarker for the presence of oral cancer, we evaluated whether microbial profiles of WMF reflected those of cancer tissue. The bacteriome in tumor tissue and paired WMF samples from patients with oral cancer differed significantly in terms of overall community structure and a PLS-DA plot demonstrated that bacterial profiles tended to cluster separately (Fig. 7A). Yet the overall composition of various taxa remained similar between the two sets of samples at phylum and genus level (Fig. 7B,C). The PCA plot for the groups is illustrated in Fig. 7D. Nevertheless, statistically significant differences between bacterial communities of WMF and tissue were still detected: abundances of four taxa belonging to the genera *Streptococcus, Lactobacillus and Bacteroidales* (Fig. 8A) and acetoin biosynthesis pathway were significantly enriched in the tissue (P < 0.01) (Fig. 8B).

### Microbial diversity

**Figure 9:**
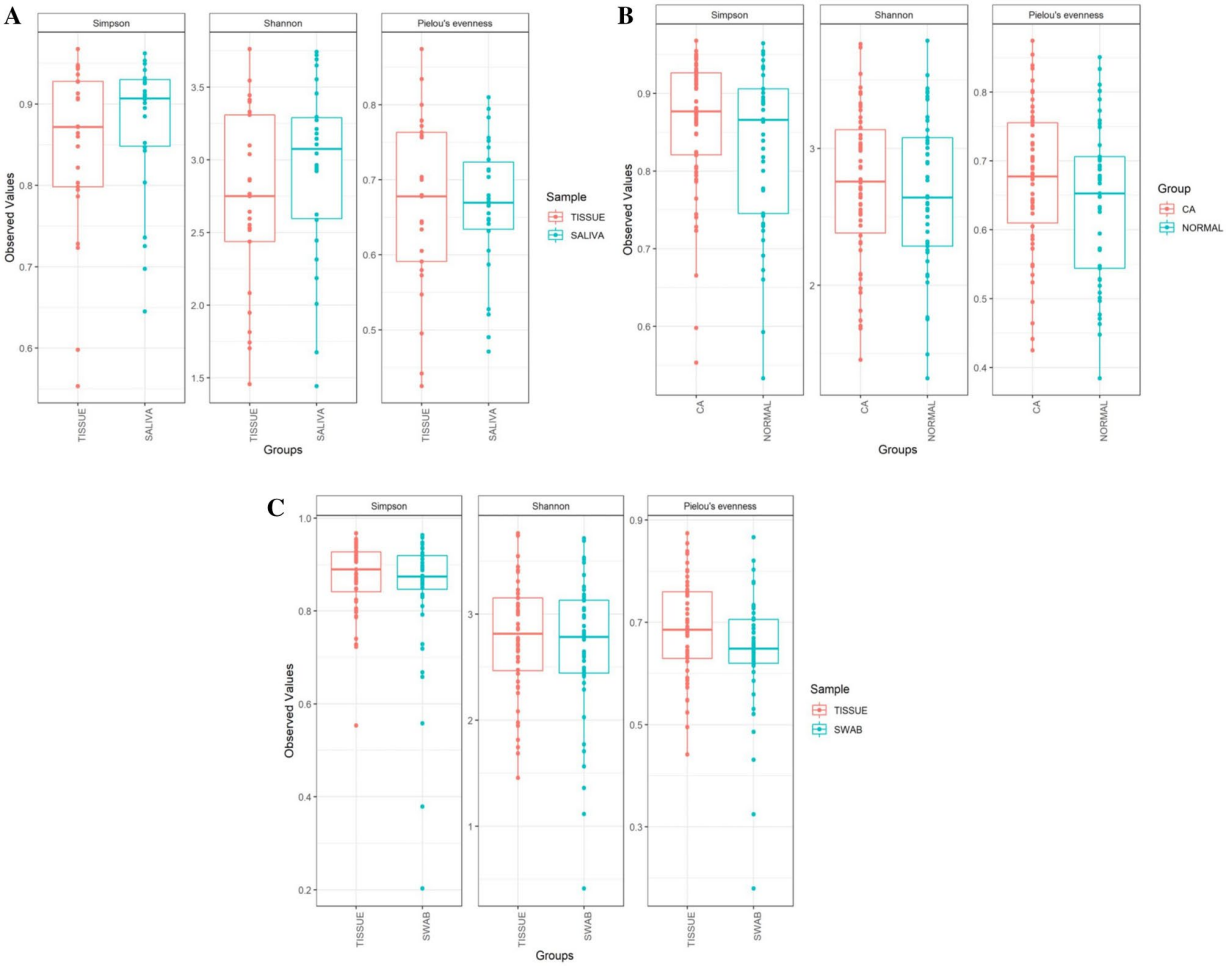
Box plot illustrating alpha diversity indices. No statistically significant difference in diversity was observed between the groups compared (P > 0.01) (**A)** CA vs controls, (**B)** tissue vs swab, (**C)** tissue vs WMF.

Shannon and Simpson indices were employed to evaluate the differences in alpha diversity. We could not detect any statistically significant difference between cancer tissue and normal tissue (P > 0.01) (Fig. 9A). Furthermore, Pielou’s evenness index, which is used to measure the evenness in a bacterial community, did not demonstrate any difference between the two groups. No difference in diversity was identified between the tumor tissue, the surface swab of the tumors or WMF (Fig. 9).

## Discussion

This study supports recent evidence that the oral bacteriome of patients with oral cancer differs considerably from that of healthy mouths^31^: these variations are not restricted to oral cancer tissues. We used MiSeq sequencing to compare the bacterial communities of oral cancer patients to those of normal healthy controls in Indian cohorts and found significant variation in the bacterial flora in WMF, tumor tissue and the tumor surface. We further demonstrated that the composition of the bacteriome sampled from within the tumor tissue differed from matched tumor surface, as well as from WMF: these are original findings. We have determined the metabolic propensity of the oral cancer bacteriome revealing significant differences in metabolic pathways between surface-adherent bacteriome and deep tissue-associated bacteriome in oral cancer patients.

Increases in the abundance of *Prevotella, Campylobacter*, *Capnocytophaga, Solobacteria, Peptostreptococcus* and *Catonella* in our patient cohort are consistent with the report by Zhao et al.in Chinese cohorts^32^. The consistency of our data relating to these genera is remarkable considering the complex nature of the human microbiome and the distinction in population characteristics, sampling techniques and methodology employed for sequence analysis across these two studies. Amongst the differentially abundant bacteria, elevated in Indian and Chinese cohorts, *Prevotella* could be speculated to have some role in cancer progression as higher levels of this genus have been reported by several others in colorectal cancer^33,34^, gastric cancer^33^ and oral cancer previously^31,34–36^. Higher abundances of *Prevotella* have also been associated with modulation of genes associated with the immune-inflammatory response in colorectal cancer^37^. Moreover, short-chain fatty acids production by *Prevotella* has been linked to hyper-proliferation of cells in both colorectal and esophageal cancer^38^. *Campylobacter*, another genus enriched in both cohorts, has been associated with esophageal adenocarcinoma in several studies and is suggested to play a role in the progression of esophageal adenocarcinoma similar to that of *Helicobacter pylori* in stomach cancer^39–41^. Bacteria of these genera have been shown to attach and invade human epithelial cells and macrophages, thereby transgressing epithelial barrier integrity and evading the host immune response^36,39,41^. Genus *Peptostreptococcus* and Genus *Capnocytophaga,* overrepresented in both populations, have been reported to be associated with oral cancer^35,36,42–44^. *Peptostreptococcus* has been shown to induce TLR2 and TLR4 expression in colon cancer cells in culture, thus boosting levels of reactive oxygen species and cell proliferation^45^. Furthermore, increases in the abundance of this genus have been reported in faecal samples from colorectal cancer patients by several authors^7,33,46–48^. The over-representation of these bacteria in multiple oral cancer cohorts expands the current knowledge on bacterial association in OSCC and further increases their significance as potential biomarkers for oral cancer across the globe. The association of *Solobacteria* in OSCC is reported here for the first time: interestingly, this genus is usually identified in association with halitosis and poor oral hygiene^49,50^ both of which are common in oral cancer patients ^5^ and so is not unexpected.

Understanding differentially abundant taxa in the mouth may help in identification of biomarkers for oral as well as other cancers. This may also inform use of antibacterial drugs in cancer prevention or treatment. However, to understand their role in carcinogenesis and in progression of a neoplasm it is important to unravel the physiological characteristics of the microbial consortia. Here, we have observed a dysbiotic microbial metabolome in oral cancer tissues. The role of oral bacteria in ethanol degradation has been well established^51,52^. Acetaldehyde is the proximal metabolite and has been classified as a Group 1 carcinogen by the International Agency for Research on Cancer of the World Health Organization^53,54^. Higher levels of acetaldehyde in WMF have been previously associated with greater risk for cancers of the upper aero-digestive tract^55,56^. Our data also illustrate an increase in the dissimilatory nitrate reduction pathway, viz: nitrate/nitrite ammonification. Nitrate reduction by bacteria has been known to vary upon oxygen tension, pH, nitrate concentration, and microbial genetics^57^. When the homeostasis is disturbed in a hostile environment, selective pressures on the microbiota can lead to the production of metabolites such as nitrites and ammonia which have the potential to promote cancer cell growth^57–61^. There exists a potential for endogenous creation of nitrosamines from nitrite in the acidic microenvironment of a carcinoma, a compound which is speculated to promote cancer progression^62–64^ and has been associated with esophageal cancers^65,66^, gastric and bowel cancers^67–69^. Phosphatidylcholine, a major eukaryotic membrane phospholipid, is present in only 15% of bacteria and is regarded as a virulence factor via interactions with eukaryotic cells of their hosts^70,71^.

Many pathways upregulated in the bacteriome associated with cancer tissues are related to degradation of fatty acids. Short-chain fatty acids (SCFA) have been shown to upregulate the production of cytokines, thereby creating a pro-inflammatory milieu in bacterial vaginosis^72^; contrariwise, they have been established as anti-inflammatory and proapoptotic in the gut^73,74^. They have been shown to affect immune responses of intestinal epithelial cells by modifying TLR-induced inflammation through the inhibition of histone deacetylases^66^. There is evidence of association of colorectal cancer with lower levels of SCFA in the tissues^75^ and similar mechanisms may well occur in the mouth.

We have also found that the structure and function of the bacteriome on the oral surface of oral cancers differs significantly from that within the tumor. The surface has a lower abundance of phylum *Bacteroidetes* and higher abundance of *Fusobacteria*. These differences are characteristic of oral mucosal biofilms, including those in periodontal pockets: the fimbriae of *Porphyromonas* and co-aggregation properties of *Fusobacterium* are well described^76–78^. *Porphyromonas gingivalis* is now regarded as a ‘keystone pathogen’ in alteration of the immune response and in triggering microbial dysbiosis within gingival biofilms^79,80^. Oral administration of *P. gingivalis* has been shown to increase the size of chemically-induced tongue cancers in mice^13^ including when combined with *Fusobacterium*^12^. *P. gingivalis* and *F. nucleatum* stimulate NF- κB signalling through Toll-like receptors and thus proliferation of human oral cancer cell lines in vitro^12^. Studies have reported increased abundance of *Fusobacteria* on the tumor surface and in WMF samples of head and neck cancer patients^14,81^. *Fusobacteria* are known for co-aggregation properties and become immobilized onto an oral surface^82,83^. Increase in abundance of *Fusobacteria* in our tumor surface samples might reflect this property.

Overall, the most abundant pathways on the tumor surface were those related to fatty acid biosynthesis, carbon metabolism, amino acid metabolism, carbohydrate metabolism, organic and polymer degradation. Increase in fatty acid biosynthesis has been demonstrated as the mechanism through which *Porphyromonas* promotes oral carcinogenesis in animal models^13^. The bacterial communities residing on the surface of the tumor are constantly exposed to the oral environment and therefore, a substantial portion of variation observed between superficial tumor and cancer tissue bacteriome may be related to WMF constituents and availability of oxygen^84^.

The bacteriome of WMF can reflect microbial communities of the oral cavity as a whole, as both hard and soft tissue surfaces are constantly bathed in WMF, from which organisms are detached by normal movements within the mouth^85^. Some recent studies have compared the microbiome of WMF in oral cancer patients to control subjects^42,86,87^ but no comparisons between WMF microbiome and cancer tissues has, to our knowledge, been published. Our data illustrate that the composition of the various phyla and genera in WMF of OSCC patients remained similar to each other at taxonomic and metabolic levels except in the elevated abundances of *Streptococcus, Lactobacillus* as well as *Bacteroides* and acetoin biosynthesis respectively. However, it should be noted that the communities associated with WMF and tumor tissue were set apart significantly in the PLS-DA plot. Our data suggest that the WMF bacteriome does partially reflect the tissue bacteriome and hence has the potential to be developed as a biomarker for prediction of disease status and prognosis.

It is important to acknowledge that functional assessments of metabolomes presented here employ a prediction method; even though validated and robust, it is based on metabolic inference from complete genomes. Also, as with every DNA based approach, the presence of that genome in the sample may not necessarily reflect whether it is active or not. Even though our study illustrates patterns of bacteriome association with oral cancer in terms of composition and function, longitudinal studies focusing on determining the causal associations of bacteriome in oral cancer are warranted. Other limitations of this study include the pooling of subjects from two different states in India and the pooling of oral subsites where the cancers were located. Further, our methods reveal only bacteria and it is well known that certain fungi and viruses contribute to oral carcinogenesis, discussion of which is outside the present work. A metagenomic approach, with “shotgun” sequencing of samples is a desirable way forward and is likely to be attempted by us and others as costs reduce.

## Conclusion

A comprehensive characterization of the bacteriome associated with surface and deep portions of oral cancer tissue, as well as the WMF in oral cancer patients, has identified specific and different microbial communities. This was manifested both in compositional as well as functional profiles of the communities. Selection of the principal source of microbial DNA is key to ensure reliable, reproducible and comparable results. Our metabolic inference to define community and metabolic structure is a novel approach for exploring the inter-bacterial relationships in the community. Nevertheless, the possibility that the observed effect might be a result of adaptation to an altered tissue microenvironment in the neoplasm itself, cannot be excluded. Combining microbial compositional profiles with metabolic profiles in prospective studies to investigate temporal orders of events in bacterial colonization in an oral cancer ecosystem would make it possible to investigate whether metabolic functionalities are significantly predictive of initiation and/or progression of these neoplasms.

## Patients and methods

### Ethics approval

This study was approved by the DY Patil Dental College Hospital, Pune (DYPY/EC/74/17), Acharya Harihar Regional Cancer Centre, Cuttack (068-IEC-AHRCC) and University of Hong Kong (UW 17-242) Ethics Review Boards. Written informed consent was obtained from all participants and all methods in this study were performed following the relevant guidelines of the Declaration of Helsinki on biomedical research involving human subjects.

### Study subjects

The study subjects were recruited from DY Patil Dental College and Hospital, Pune and Acharya Harihar Regional Cancer Centre, Cuttack in India. Controls were matched for age and gender. We did not include smoking and other habits for matching because of the within the time frame.

The final inclusion criteria for the cancer cases were:

- Diagnosis of pathologically confirmed squamous cell carcinoma of mucosal tongue, gum, floor of the mouth, palate, and other mouth, corresponding to the International Classification of Diseases, 10th revision [ICD-10] rubrics C02-06.
- No previous cancer diagnosis
- No history of antibiotic use in the past one month
- Adults ready to provide informed consent.

The inclusion criteria for the controls were:

- Patients reporting to the institution for removal of asymptomatic third molars
- Absence of any signs and symptoms of local infections in the oral cavity
- Absence of any potentially malignant disorders
- No previous cancer diagnosis
- Age and gender-matched adults ready to provide informed consent.

### Sample collection

Fluid sampled from the mouth is predominantly the secretions of the major and minor salivary glands but includes mucosal transudate, a variable inflammatory exudate and a significant contribution from the gingival crevicular fluid. We prefer, therefore, to refer to samples taken from the mouth as whole mouth fluid (WMF). WMF and swab samples were collected from the oral cavity of patients after a provisional clinical diagnosis of oral cancer by a specialist at the initial appointment but prior to biopsy, surgery, radiotherapy, or chemotherapy. However, the final inclusion of the sample into the study was only after histopathologic confirmation of the case by an experienced pathologist. Each subject should have refrained from smoking, chewing, drinking, or eating for at least 30 min before the sample collection^25,42^. A swab of the surface of the oral cancer lesion was collected using Isohelix Swab SK-2S (Isohelix, UK) to collect superficial cells. The 2K-2S swab collection harbors a unique cap design which helps in minimizing contamination during sample collection and handling at the DNA isolation stage which is very critical for microbiome studies. The swab was rubbed over the cancer lesion multiple times firmly for 1 min and immediately stored in – 80 ∘C freezer. WMF was collected by GeneFix saliva Collection device-1 ml (Isohelix, UK) according to manufacturer’s instruction. The device is prefilled with stabilization buffer (1 ml) which helps in preserving DNA at room temperature and comes with a simple screw-on funnel to facilitate collection. The subjects were asked to spit into the funnel attached to the tube until the 2 ml line (one ml of saliva is collected). The WMF collection devices were stored at room temperature until DNA extraction. Tissue samples were removed from the body of the tumor during surgery and were immediately rinsed in normal saline and stored in a prefilled vial containing 2 ml of nucleic acid stabilizing solution RNA*later* (Invitrogen, USA). Special precautions were taken to avoid the surface of the tumor. The vial was stored in 4 ^∘^C freezer for 24 h before transferring to – 80 ∘C freezer to give enough time for the solution to penetrate tissues. Patients undergoing third molar extractions that were asymptomatic were selected as controls. Retromolar tissue of ~ 0.5 cm^3^ was excised and handled in the same way as the cancer tissues.

### Sample processing and sequencing

The stored samples were processed at Rajiv Gandhi Centre for Biotechnology, Kerala. The swabs were thawed at room temperature and 2 ml phosphate-buffered saline (PBS) (Gibco, Life technologies, US) was added and incubated for 6 h at 4 ∘C. The tube was vortexed thoroughly for 15 s and pelleted at 7500 rpm (10 min). DNA was then extracted by QIAamp DNA Mini Kit (Qiagen, Manchester, UK) according to manufacturer’s instruction with an added step on lysozyme treatment from the Appendix Protocol “Isolation of genomic DNA from Gram-positive bacteria” from Qiagen Handbook (Qiagen, Manchester, UK). In brief, the pellet was treated with 180 μL of an enzyme solution [20 mg/ml lysozyme (Sigma Aldrich,) in 20 mM Tris–HCl (pH 8.0), 2 mM EDTA, and 1.2% Triton-X 100] at 37 °C for 1 h. The resultant solution was then processed for DNA extraction.

For the processing of the cancer tissue, deeper portions of tissue (~ 4–5 mg) were dissected from the tumor and placed immediately in a 1.5 ml Eppendorf tube and homogenized using Polypropylene micropestle (Tarson, UK). 180 μL of lysis enzyme solution was added and incubated for 1 h at 37 °C. Subsequently, Proteinase K (QIAamp DNA Mini kit, Qiagen) was added and samples were incubated at 56 °C in a water bath with periodical vortexing until total lysis of tissue was observed (~ 6 h). Samples were then incubated in 200 μL Buffer AL (QIAamp DNA Minikit, Qiagen) for 30 min at 70 °C before continuing extraction using QIAamp DNA Minikit (Qiagen). According to the manufacturer's instructions (37), the samples were then loaded on a DNA spin column (Qiagen) and centrifuged at 8000 rpm in a tabletop centrifuge. The columns were then washed with Qiagen buffers AW1 and AW2 and finally DNA eluted using the elution buffer in the kit.

WMF samples were stored at room temperature and DNA extraction was carried out according to manufacturer instructions using the Gene Fix WMF Prep 2 Isolation kit (Isohelix, UK) which was specifically optimized for the Gene Fix WMF DNA Collectors. Before Proteinase K treatment, samples were subjected to 30 min of enzymatic lysis at 37 °C with lysis buffer containing lysozyme (20 mg/ml) (Sigma-Aldrich, Dorset, UK). The extracted DNA was then stored at − 20 °C.

DNA sample integrity was assessed by electrophoresis on 1% agarose gel. The concentration of DNA in the samples was determined in a Microplate Reader (Qubit Fluorometer, Invitrogen). Amplification of bacterial DNA to create 16S libraries was performed using PCR primers targeting the 16S rRNA gene V3-V4 (319F-806R) and the products were purified with Ampure XP beads (AGENCOURT).

The primer sequence was:

341F-ACTCCTACGGGAGGCAGCAG

806R-GGACTACGTGGGTATCTAAT

After quantification by real-time quantitative PCR (RT-qPCR) (EvaGreen) the qualified libraries were sequenced on the Illumina MiSeq System using the PE300 reagent Kit. All solutions utilized in sample processing, PBS, lysozyme buffers, reagents in commercial kits and empty tubes from the isolation kit were treated as negative controls. All these underwent the respective DNA extraction procedures. Absence of contaminating bacteria was confirmed as all failed the amplification step for 16S libraries.

### Bioinformatics and statistical analysis

The quality control (QC) filter was performed using DADA2 and the amplicons were paired and clustered^88^. Phylotype and gene inference analyses were performed by first aligning the quality-controlled query reads to the reference alignment with Infernal^89^ placing them on the phylogenetic reference tree with pplacer^90^. Taxonomical classification and gene inferences were based on edge placement and consensus identity with either internal or terminal nodes as described in Bowman and Ducklow^30^. The diversity indices were constructed using Phyloseq^91^. To see structural segregation between groups, Partial Least Squares Discriminant Analysis (PLS-DA) model was generated using PRIMER7^92^. Functional metagenome of the oral microbiome based on BioCyc orthology abundance was inferred using PAPRICA and VOOM^93,94^. Differential abundances of various edges as well as functional pathways, were analyzed by DESeq2^95,96^.

## Supplementary Information

Supplementary Dataset 1.

Supplementary Dataset 2.

Supplementary Dataset 3.

Supplementary Information.
