# Supplementary material for: Differences in the bacteriome of swab, saliva, and tissue biopsies in oral cancer
Source: Sci Rep. 2021 Jan 13;11:1181. doi: 10.1038/s41598-020-80859-0 (PMC7806708; doi:10.1038/s41598-020-80859-0)
Supplement: Supplementary file 4 — Supplementary Information. [file 41598_2020_80859_MOESM4_ESM.docx]

SUPPLEMENTARY DATA

**The bacteriome of oral cancer in India: Compositional and metabolic differences in swab, saliva, and tissue biopsies**

Divya Gopinath, Rohit Kunnath Menon, Chong Chun Wie, Moinak Banerjee, Swagatika Panda, Deviprasad Mandal, Paresh Kumar Behera, Susanta Roychoudhury, Supriya Kheur, Michael George Botelho, Newell W. Johnson

| **Parameter** | **OSCC** | **Normal controls** |
| --- | --- | --- |
| **Total no of subjects** | 48 | 46 |
| **Age, mean (years)** | 49.31 | 50.67 |
| Standard deviation SD | ±13.24 | ±6.81 |
| **Gender** |  |  |
| Male | 35 | 35 |
| Female | 13 | 11 |
| **Tobacco Habits** |  |  |
| Smoking | 6 | 14 |
| Chewing | 18 | 15 |
| Chewing + Smoking | 24 | 16 |
| **Alcohol Habit** |  |  |
| Yes | 37 | 14 |
| No | 11 | 32 |
| **Anatomic sites** |  | NA |
| Floor of mouth | 3 |  |
| Buccal Mucosa | 13 |  |
| Lateral Border of tongue | 9 |  |
| Dorsal tongue | 6 |  |
| Alveolus | 10 |  |
| Gingiva | 7 |  |
| **Clinical Staging** |  | NA |
| Lymph node + | 39 |  |
| Lymph node – | 9 |  |
| **Histological Grade** |  | NA |
| Well | 30 |  |
| Moderate | 16 |  |
| Poor | 2 |  |

**Supplementary Table 1 :** Clinicopathological characteristics of the samples


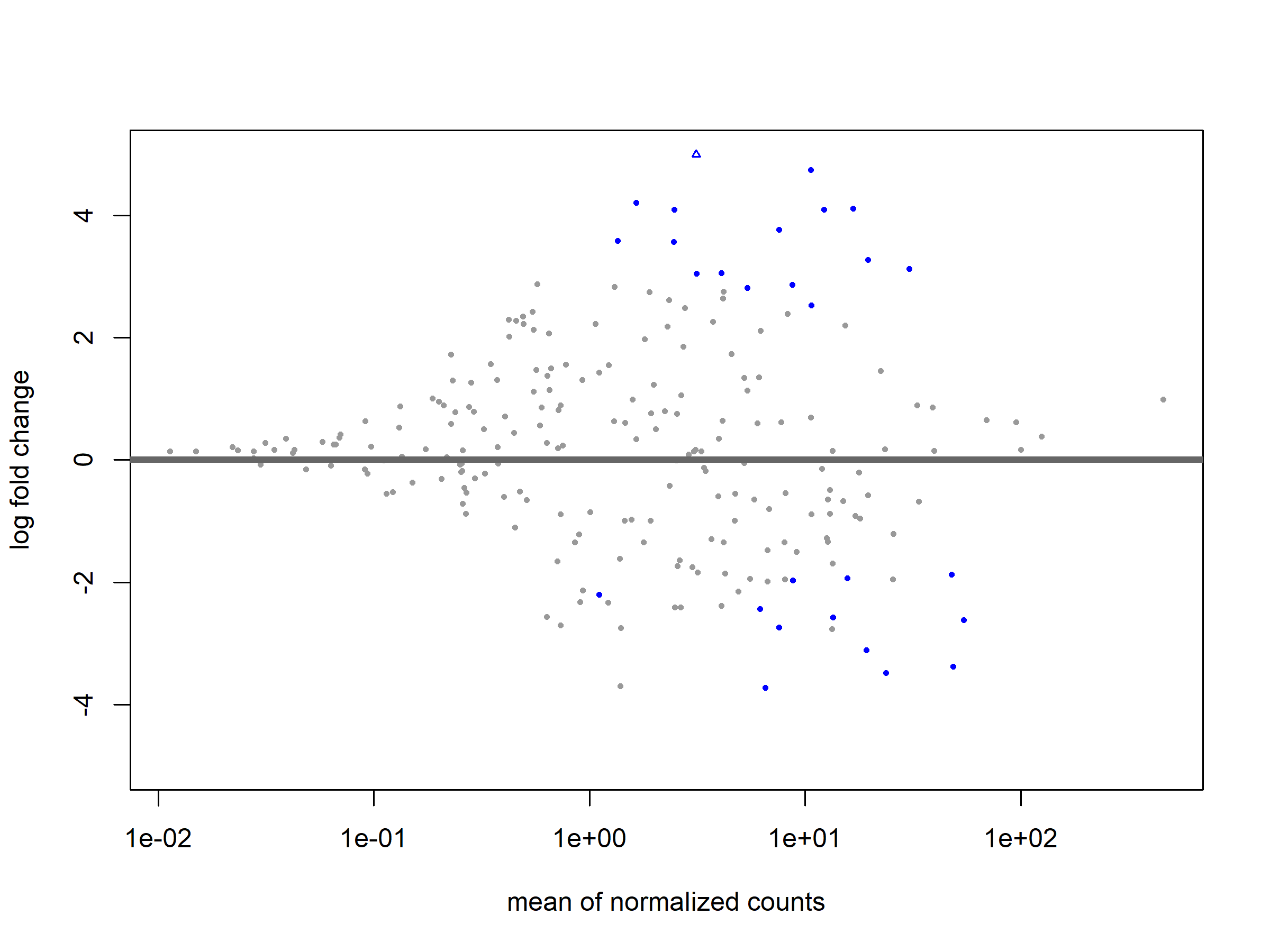


Supplementary figure 1: Supporting MA plot for Figure 2


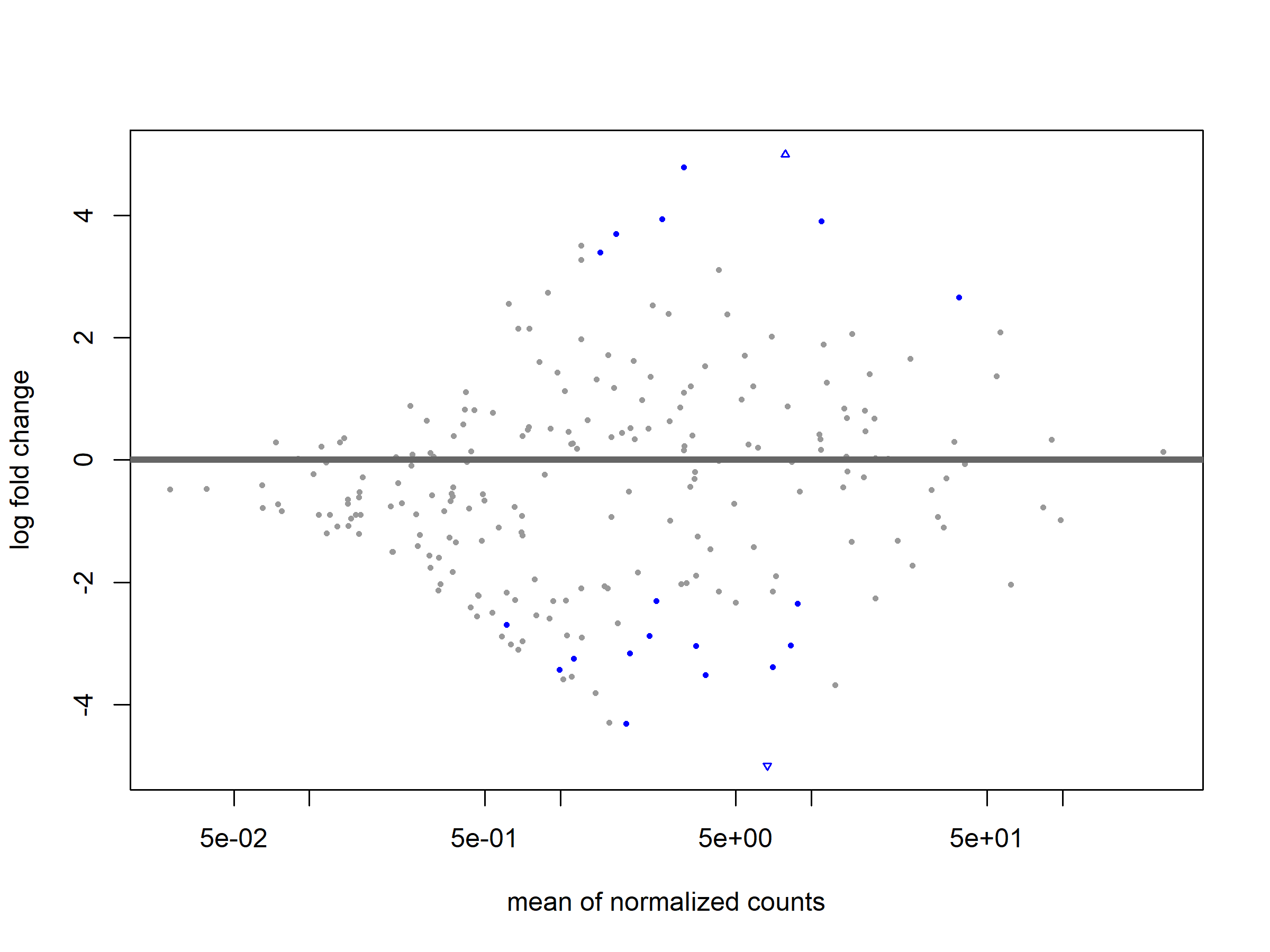


Supplementary figure 2: Supporting MA plot for Figure 5


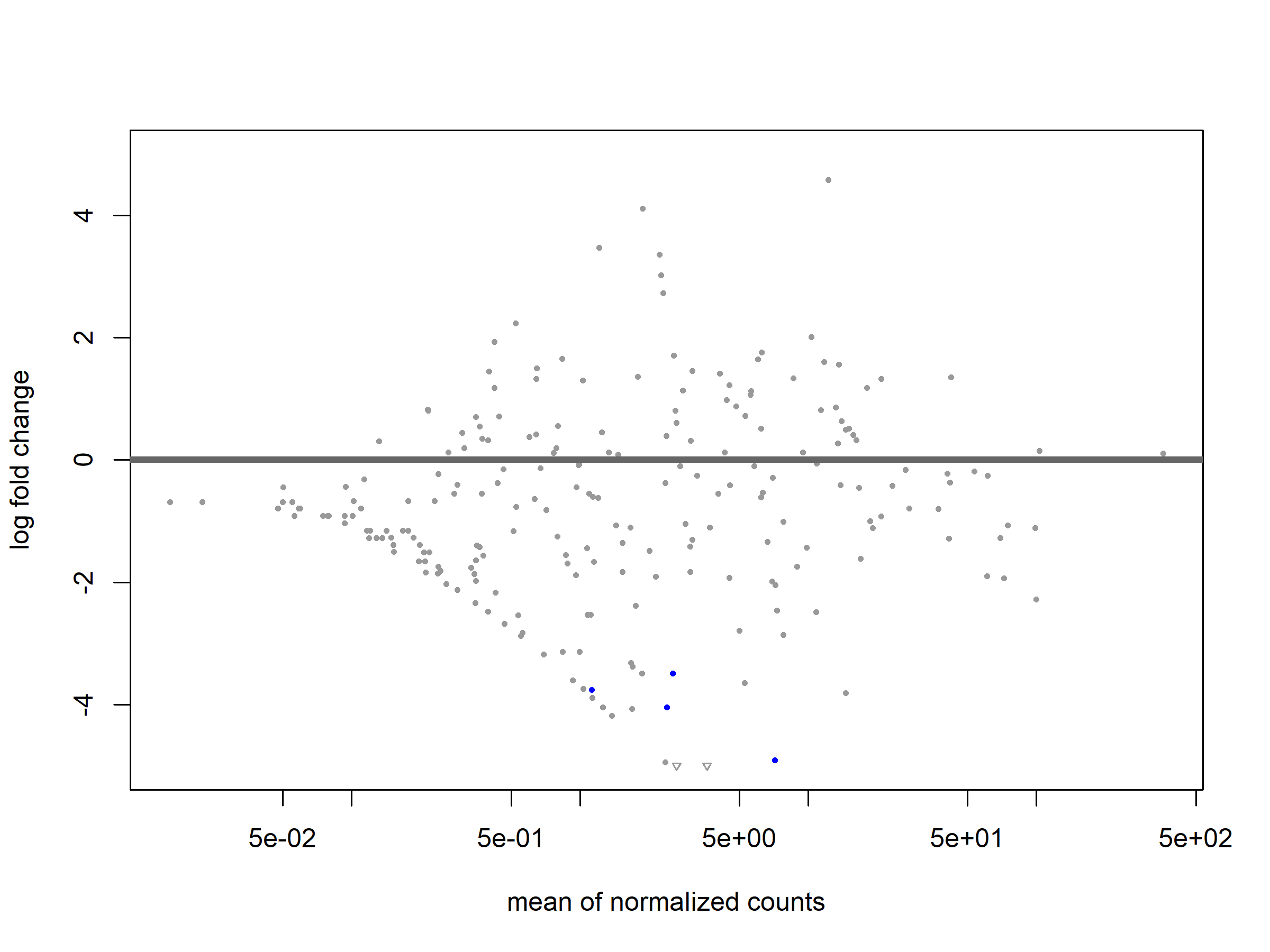


Supplementary figure 3: Supporting MA plot for Figure 8
